# Partially Ablative Radiotherapy for Bulky Tumors: A Narrative Review of a Developing Concept

**DOI:** 10.3390/jpm15110533

**Published:** 2025-11-03

**Authors:** Savino Cilla, Costanza Maria Donati, Milly Buwenge, Gabriella Macchia, Francesco Deodato, Silvia Cammelli, Alessio Giuseppe Morganti

**Affiliations:** 1 Medical Physics Unit, Responsible Research Hospital, 86100 Campobasso, Italy; 2 Radiation Oncology Unit, Istituto di Ricovero e Cura a Carattere Scientifico Azienda Ospedaliero-Universitaria di Bologna, 40138 Bologna, Italymilly.buwenge@unibo.it (M.B.); alessio.morganti2@unibo.it (A.G.M.); 3 Radiation Oncology Unit, Responsible Research Hospital, 86100 Campobasso, Italyfrancesco.deodato@unicatt.it (F.D.); 4 Radiology Institute, Università Cattolica del Sacro Cuore, 00168 Roma, Italy; 5 Department of Medical and Surgical Sciences, Alma Mater Studiorum University of Bologna, 40138 Bologna, Italy

**Keywords:** partial ablative radiotherapy, bulky tumors, palliative radiotherapy

## Abstract

The management of large bulky tumors is very challenging. The current treatment options for effective palliation of symptoms are limited. These tumors often present a large burden at the time of diagnosis, growing along critical bony and neural structures and preventing surgical resection in most of the cases. These tumors are also known to be relatively resistant to chemotherapy, with very low response rates. In addition, conventional photon-based radiotherapy has a limited effect due to their radioresistance, the use of large treatment fields, and the impossibility of delivering high doses because of the higher risk of normal tissue toxicity. Therefore, more effective radiation treatments for palliation are needed to achieve greater local control rates. A recent approach called partial ablative radiotherapy (PART) has been shown to be potentially able to improve the effectiveness of radiotherapy. This technique is based on the ability of recent advanced delivery techniques to deliver a high “ablative” dose to the central part of the tumor, maintaining a very low and safe dose profile at the periphery to spare the surrounding organs at risk. Although this technique has been evaluated only in small studies and case reports, it showed notable treatment responses and safety profiles. The present narrative review describes the rationale for PART, the current and forthcoming state of evidence, the existing studies, and the future directions for the development of this approach, including the associated challenges.

## 1. Introduction

Bulky tumors are unresectable tumors of unusually large size and volume, usually associated with adverse features such as aggressive biology, fast growth, presence of a large hypoxic component, radioresistance, and close relationship with surrounding normal tissues. These multiple unfavorable disease characteristics make its treatment extremely difficult and consistent only with palliative or best supportive care. Partially ablative radiotherapy (PART) is an emerging strategy in radiation oncology for the treatment of bulky tumors [1]. It involves delivering ablative doses of radiation to a defined portion of the tumor, rather than the entire mass. This approach aims to balance the potential for high local control with the need to minimize treatment-related toxicity and preserve patient quality of life. The rationale for PART is based on several key observations. First, not all parts of a bulky tumor contribute equally to its malignant behavior. Targeting the most aggressive or symptomatic areas with ablative doses may be sufficient to achieve meaningful clinical benefit. Second, in palliative settings, where the goal is symptom control rather than cure, partial ablation can offer a less toxic alternative to whole-tumor irradiation. Third, PART may have a role as a neoadjuvant therapy, potentially downsizing the tumor before surgery and improving resectability.

Several studies have explored the feasibility and efficacy of PART in various tumor types, including non-small-cell lung cancer [2,3,4], chordomas [5], sarcomas [4,6,7,8,9], head-and-neck cancer [4], hepatocellular cancer [10], breast cancer [11], and urinary tract [12] and rectal malignancies [13]. These studies, along with others related concepts like stereotactic body radiation therapy (SBRT) [14], spatially fractionated radiotherapy (SFRT) [15], and “lattice” radiotherapy (LRT) [16], provide a foundation for understanding the potential of PART. SBRT involves the delivery of very high individual doses of radiation in few fractions to small tumors or oligometastases in various extracranial sites with high precision and accuracy, capable of achieving high local control [15]. On the other hand, large tumors, which also require high doses to be controlled due not only to their dimensions, but because of the presence of necrotic and hypoxic areas, cannot be treated with SBRT due to the risk of major complications and toxicities. SFRT, and more recently its three-dimensional evolution, known as LRT, have been proposed as techniques able to deliver radiation doses within the target volume in a heterogeneous pattern with regions of peak and valleys of over- and underdosage, aiming to provide timely symptom relief maintaining treatment toxicity within an acceptable range.

In this scenario, there is an unmet need to provide more effective palliative treatments for unresectable bulky tumors. PART represents an interesting strategy to fill the gap present in large lesion management, delivering very high doses to the tumor core in order to achieve a durable tumor response while accepting low doses to the tumor periphery to spare the adjacent normal tissues. In addition, PART could be combined with other treatment modalities, such as chemotherapy or immunotherapy, to enhance overall treatment efficacy.

This narrative review delves into the specifics of PART, examining the rationale, techniques, clinical evidence, challenges, and future directions of this emerging approach. We also analyze the available clinical data, focusing on outcomes such as local control, survival, and toxicity. Finally, we discuss the challenges and open questions that remain in the field, highlighting areas for future research.

## 2. Materials and Methods

This narrative review was based on a comprehensive search strategy on the PubMed and Scopus databases for the following indication terms, combined with the Boolean operators AND and OR: partial ablative radiotherapy, PART, partial tumor irradiation, modified simultaneous integrated boost, partially ablative body radiation therapy, tumor core boost, bulk tumor radiotherapy, bulky tumors, radiotherapy strategies, dose escalation, partial radiotherapy, partial tumor targeting, partial tumor ablation, selective radio-therapy, radiotherapy for bulky tumors. The literature search was conducted on December 2024 for only full-text articles in English, although the research strategy did not strictly follow the criteria for a systematic review. Also, an exhaustive study of the reference section of each article included herein was carried out.

Considering the limited amount of available information in the literature, we included all types of publications in the search, including case reports. The final analysis included 23 publications.

## 3. Rationale and Clinical Applications

Partial ablative radiotherapy addresses the limitations of conventional radiotherapy in managing bulky tumors, where treating the entire volume with ablative doses can lead to substantial toxicity, especially when critical organs are nearby. PART offers a targeted approach, concentrating the highest doses on the most clinically relevant areas while minimizing exposure to surrounding healthy tissues. This approach becomes particularly advantageous in the following clinical scenarios.

### 3.1. Palliation of Symptoms

When a bulky tumor causes localized symptoms like pain, bleeding, or obstruction, PART can target the symptomatic area for effective relief without the burden of whole-tumor irradiation. This approach prioritizes improving the patient’s quality of life by addressing the most pressing clinical issues. The role of palliative radiotherapy in a changing oncologic landscape is today well considered, especially highlighting the importance of symptom control [17].

### 3.2. Combined Modality Therapy

PART could be combined with other treatment modalities, such as chemotherapy or immunotherapy, to enhance overall treatment efficacy. The localized ablative effect of PART may synergize with systemic therapies, leading to improved outcomes. In particular, it is nowadays quite clear that radiotherapy, in addition to its well-established tumoricidal effects, is able to activate the host immune system by modulating tumor phenotypes, enhancing antigen presentation and tumor immunogenicity, boosting the production of cytokines, and altering the tumor microenvironment. The integration of modern radiotherapy with immunotherapeutic agents, also promoting the host antitumor immune response, has been an obvious step. In recent years, new data suggested that radiation-induced immune responses might be dose-dependent [18]. Therefore, with the widespread adoption of ablative radiation doses, the question of a further enhancement of these effects by combining immunotherapy and stereotactic ablative radiotherapy was investigated on the basis that ablative doses, in addition to direct cell killing, might encourage these killed cells to act as a vaccine in situ [16]. Currently, a few clinical trials of this approach are ongoing in patients with advanced solid tumors [19].

## 4. Strategies for Treatment Planning

Treatment planning for PART requires careful consideration of several factors, including target volume definition, dose constraints for surrounding organs at risk, and the choice of radiotherapy technique. Advanced imaging modalities, such as CT, MRI, and PET, are essential for accurate target delineation and treatment planning. Furthermore, image guidance during treatment delivery can help ensure accurate dose delivery and minimize interfractional variations. PART can be delivered using various radiotherapy techniques and strategies, each with its own advantages and limitations. Usually, a combination of the following treatment techniques for planning and delivery allows for optimization of a balance of accuracy, dose intensification, and patient safety.

### 4.1. Stereotactic Body Radiotherapy (SBRT)

Stereotactic body radiation therapy (SBRT) is a precise form of radiation therapy that delivers high doses to small tumor lesions over a shorter treatment period compared to traditional radiotherapy while limiting the dose to the surrounding organs. This treatment strategy can be partially applied/used in PART for targeting well-circumscribed volumes within a large tumor mass with ablative doses in few fractions while treating the remaining part of the tumor, closer to the normal tissues, with tolerable doses. In this way, PART can be seen as an optimized palliative approach in which dose intensification only in the most aggressive or symptomatic areas with ablative doses may be sufficient to achieve meaningful clinical benefit in terms of symptoms control. In this context, a few recent studies have made noteworthy contributions to the exploration of partial-volume SBRT, particularly within the context of reirradiation [20,21].

### 4.2. Hypofractionated Radiotherapy

This technique involves delivering higher doses per fraction over a shorter overall treatment time compared to conventional radiotherapy. This is a common approach in radiotherapy and can be adapted for PART by delivering ablative doses to the targeted area while reducing the overall treatment burden for the patient.

### 4.3. Intensity-Modulated Radiotherapy (IMRT)/Volumetric Modulated Arc Therapy (VMAT)

IMRT allows for precise dose sculpting, enabling the delivery of high doses to the target volume while sparing adjacent critical structures. This is particularly important in bulky tumors, where critical organs may be in close proximity.

## 5. Clinical Evidences

The clinical evidence for PART is still evolving, with ongoing research exploring its efficacy and safety in various tumors types. While randomized controlled trials are still lacking, several prospective and retrospective studies have demonstrated promising results.

### 5.1. Lung Cancer

Bai et al. [2] reported on a retrospective study of partial SBRT in bulky non-small-cell lung cancer, showing promising local control rates and survival outcomes. Thirty patients were treated with one partial SBRT plan (5–9 Gy per fraction, three to six fractions) to the gross tumor boost, followed by one standard plan to the planning target volume. Eleven and ten patients had partial response and stable disease, respectively. Two-year local control and overall survival were 85.7% and 55.6%, respectively. No severe acute side effects above grade 3 were observed. This study suggests that PART may be a viable option for patients with bulky lung tumors who are not candidates for surgery or conventional radiotherapy. Tubin et al. [3] evaluated a novel SBRT-based partial tumor irradiation (SBRT-PATHY) to enhance the radiotherapy therapeutic ratio of advanced lung cancer. Twenty patients considered inoperable or unsuitable for radical radiochemotherapy were enrolled and treated with one to three fractions each of 10–12 Gy to the tumors. These patients (group 1) were compared with recommended standard-of-care chemotherapy (group 2) and institutional conventional palliative radiotherapy (group 3). One-year overall survival was 75%, 60%, and 20% in groups 1, 2, and 3, respectively. The 1-year cancer specific survival was 90%, 60%, and 20% in groups 1, 2, and 3, respectively. The bulky tumor control rate was 95% for SBRT-PATHY compared to 20% in the other two groups. In particular, significant bystander and abscopal effects were seen by SBRT-PATHY in 95% and 45% of patients, respectively.

### 5.2. Head-And-Neck Cancer

Wittgenstein et al. [4] reported the results of a prospective study using a pragmatic approach of a simultaneously integrated boost to a geometrically defined tumor core, aiming to deliver a minimum dose of 150% of the prescribed dose to the gross tumor volume tumor core and to reach a maximum of at least 200% in the tumor core. Of the 21 enrolled patients, 7 had tumors located in the head-and-neck region. Treatment delivery and short-term follow-up was successful in all patients. The high doses applied, up to 50 Gy in five fractions (or 60 Gy in ten fractions), did not cause unexpected side effects in the follow-up period.

### 5.3. Sarcomas

Locally advanced, bulky, unresectable sarcomas cause significant tumor mass effects, leading to burdensome symptoms. Shah et al. [7] explored in a recent review article the evolving role of radiotherapy in metastatic soft tissue sarcoma from palliation to ablative strategies. The authors underlined the potential of SBRT to offer a safe, convenient, precise, and non-invasive option for ablation of sites of metastases. Yu et al. [9] reported the clinical implementation of a novel partially ablative body radiotherapy (PABR) technique able to deliver high ablative doses to the tumor core and low palliative doses to its periphery, aiming to increase overall tumor response without significantly increasing treatment toxicity. A total of 18 patients with bulky, unresectable sarcomas treated with PABR were retrospectively reviewed. The most common regimen used was 20 Gy in five fractions with an intratumoral boost dose of 50 Gy. After a median follow-up of 11 months, 89% of patients exhibited a partial response with a mean absolute tumor volume reduction of about 50%. All symptomatic patients experienced symptom improvement. One-year overall survival and freedom from local progression were 61% and 83.3%, respectively, without any grade 3 or higher toxicities. Nomiya et al. [8] reported the case of a large retroperitoneal tumor treated with modified simultaneous integrated boost radiotherapy unable to undergo surgery. Modified SIB radiotherapy was administered with a dose to the center of the tumor and to the surrounding healthy tissue of 96 Gy and <60 Gy, respectively, in 33 fractions. At the end of the treatment, the tumor volume was reduced by ≥80%, and the residual tumor was surgically resected. As a result of the resection, a complete pathological response was confirmed. The patient has been recurrence-free >3 years with no complications. Medici et al. [6] reported a case of complete metabolic response after partially ablative radiotherapy for a bulky, rapidly growing, undifferentiated retroperitoneal liposarcoma in an old patient, where surgical and systemic therapies were ruled out due to age and comorbidities. PAR PART was administered using VMAT, delivering 20 Gy in four fractions twice daily to the macroscopic tumor and 40 Gy in four fractions twice daily to the central part of the tumor. An 18F FDG–PET–CT scan performed after radiotherapy demonstrated a complete metabolic response throughout the entire tumor mass. Although the patient eventually succumbed to metastatic spread to the bone, liver, and lung after 9 months, no local disease progression or pain/obstructive symptoms were observed.

### 5.4. Chordomas

Cilla et al. [5] investigated the application of PART for a large-mass chordoma tumor using a simultaneous integrated boost. A modified SIB treatment was implemented to irradiate the central volume of the tumor up to 10 Gy/fraction in a dose escalation trial while maintaining the remaining tumor volume and the surrounding healthy tissues within 5 Gy/fraction in twice daily fractions for two consecutive days. The authors showed the feasibility of dose escalation to the central portion of the tumor while maintaining an acceptable risk of toxicity.

### 5.5. Hepatocellular Cancer

Lin et al. [10] reported a complex case of an elderly patient with unresectable bulky hepatocellular carcinoma with co-infections of hepatitis B and C virus. The patient referred to radiotherapy because no alternative treatment (surgery, radiofrequency ablation, or transcatheter arterial chemoembolization) was recommended because of their age. Radiotherapy was delivered in 26 fractions of 39 Gy, 52 Gy, and 57 Gy to the outer target, middle target, and inner target volume. After radiotherapy, a large tumor volume reduction rate of 89% was observed (from 608.4 c.c. to 68.7 c.c.). Significant symptom alleviation and tumor volume reduction were observed for 32 months until multiple bone metastases arose. No severe treatment toxicity was noted during or after RT.

### 5.6. Urinary Tract Malignancies

Chen et al. [12] recently presented an update of clinical outcomes and dosimetric analysis of PART in 26 patients with inoperable locally advanced bulky urinary tract malignancy. PART treatments consisted of a dose of 15–32 Gy in three to five fractions to the gross tumor volume boost, followed by conventional fractionated radiotherapy (40.0–58 Gy in 15–26 fractions) to the planning target volume. The authors reported local control and overall survival at 2 years of 83.2% and 45.5%, respectively. Local symptoms improved in most patients after PART. No patient was observed to experience grade 3 or higher toxicity directly induced by radiotherapy.

### 5.7. Breast Cancer

Nomiya et al. [11] reported the outcomes of three patients with macroscopic adenocarcinoma of the breast diagnosed as T4b treated with a modified simultaneous integrated boost radiotherapy technique. The median total dose to the tumor tissue part was 110–140 Gy. In all three patients, it was macroscopically confirmed that all tumors disappeared with a median time of 90 days. Local cancer pain was relieved in all patients. Although skin defects persisted because of tumor disappearance, there were no grade 3 or higher toxicities due to radiotherapy.

### 5.8. Rectal Cancer

Nomiya et al. [13] reported the case of a huge pelvic tumor diagnosed as a rectal adenocarcinoma treated with a modified simultaneous integrated boost technique. Doses of 77 Gy and 64.5 Gy were delivered to the center of the tumor and the surrounding area, respectively. The tumor, with an initial maximum size of 15 cm, disappeared four months after the start of the radiotherapy.

The literature on clinical evidences is summarized in Table 1. All these preliminary experiences highlighted the potential of PART to improve local control and symptom palliation while minimizing toxicity compared to conventional radiotherapy. By targeting the most aggressive or symptomatic areas within a bulky tumor, PART offers a more focused and potentially more effective approach. However, further research is needed to define the optimal patient selection criteria, treatment parameters, and long-term out-comes of PART in various tumor types. Larger prospective studies, ideally randomized controlled trials, are essential to establish the role of PART in the management of bulky tumors.

## 6. Bystander and Abscopal Effects

Tubin et al. [20] aimed to assess outcomes with the use of SBRT for partial tumor irradiation of unresectable bulky tumors targeting exclusively their hypoxic segment, aiming to exploits the non-targeted effects of radiotherapy in terms of bystander effects (local) and abscopal effects (distant). For twenty-three patients, the hypoxic tumor segment was defined as the hypovascularized–hypometabolic junctional zone between the central necrotic and peripheral hypervascularized–hypermetabolic tumor segment and irradiated with one to three fractions of 10–12 Gy. The pathological lymph nodes and metastases were not irradiated in order to assess the distant non-targeted effects of radiation (abscopal effect). No patient received any systemic therapy. The bystander and abscopal response rates were 96% and 52%, respectively. Median shrinkage of partially irradiated bulky tumor expressing intensity of the bystander effect was 70% (range 30%–100%), whereas for the non-irradiated metastases (intensity of the abscopal effect), it was 50% (range 30%–100%). No patient experienced acute or late toxicity of any grade.

The same research group presented the early results of novel partial bulky tumor irradiation using carbon ions and protons in eleven patients with recurrent unresectable bulky tumors who failed previous state-of-the-art treatments including radiochemotherapy [22]. A bystander tumor volume was created within the gross tumor volume and irradiated with 30–45 Gy RBE in three consecutive fractions. The peritumoral immune microenvironment surrounding the gross tumor volume, containing nearby tissues, blood–lymphatic vessels, and lymph nodes, was considered an organ at risk and protected by highly conservative constraints. With a median follow-up of 6.7 months, overall survival was 64%, and 46% of patients were progression-free. The average tumor volume regression was 61% from the initial size. The abscopal effect was observed in 60% of patients.

## 7. Challenges and Future Directions

Partial ablative radiotherapy, while promising, faces several challenges that need to be addressed to optimize its clinical implementation and broaden its applicability. Future research directions aim to overcome these challenges and refine the technique for im-proved patient outcomes.

### 7.1. Target Delineation and Heterogeneity

Accurately defining the target volume for PART can be challenging, especially in heterogeneous tumors, where the most aggressive or symptomatic areas may not be clearly demarcated. Advanced imaging techniques, such as functional imaging and image-guided biopsies, may help improve target delineation and ensure that the ablative dose is delivered to the most clinically relevant regions. Usually, the boost volume, i.e., the tumor core, lies completely inside the whole macroscopic tumor, as defined in the planning computed tomography. A few authors defined the boost volume as a geometrical shell structure inside the GTV, created with an isotropic 1–2 cm erosion from the GTV outer contour depending on tumor size and its location in relation to critical organs [4,5,9]. This distance was chosen to allow enough space for dose falloff between the two different dose levels. Other authors used a combination of CT and 18F FDG–PET to define the core volume, taking into account the different contrast-enhanced (peripheral vascularized), the contrast-unenhanced (central necrotic), and/or the contrast-hypoenhanced (hypoxic hypovascularized) regions of the tumor [20].

### 7.2. Treatment Planning

Optimization of the dose distribution can be complex, requiring advanced treatment planning techniques. The development of new automated planning strategies [23,24] may help streamline its implementation in clinical routine. In this context, recent advancements aimed to develop a biological dose prediction model considering tissue bioreactions in addition to patient anatomy for achieving a more comprehensive evaluation of tumor control and promoting the automatic planning of bulky lung cancer [25]. The predicted biological dose distribution may enable a quick intuitive evaluation of tumor ablation and the modification of the ablation range to improve BED of tumor targets.

### 7.3. Toxicity Management

While PART aims to minimize toxicity by sparing surrounding normal tissues, the high doses used can still lead to adverse effects. Careful patient selection, meticulous treatment planning, and advanced delivery techniques are crucial for minimizing toxicity and managing any side effects that may occur.

### 7.4. Treatment Response Assessment

Evaluating the response to PART can be complex, as conventional imaging criteria may not accurately reflect the biological effects of the targeted ablation. Functional imaging techniques, such as PET and fMRI, may provide more sensitive measures of treatment response and help guide further management decisions.

### 7.5. Technological Advancements

Ongoing technological advancements in radiotherapy, such as real-time image guidance, adaptive radiotherapy, and particle therapy, may further enhance the precision and efficacy of PART. These technologies can improve target localization, adapt to tumor motion and changes during treatment, and potentially reduce toxicity to surrounding tissues. Novel unconventional radiotherapy techniques, including advanced imaging and delivery methods that may contribute to the future development of PART, have been discussed by Tubin et al. [26].

### 7.6. Spatially Fractionated Radiotherapy (SFRT) and Lattice Radiotherapy (LRT)

The potential of PART could also be exploited by the application of unconventional techniques such as spatially fractionated radiotherapy (SFRT) and lattice radiotherapy (LRT). SFRT is a technique that delivers a non-uniform radiation dose distribution across the target volume using grids or sieves to create spatially fractionated dose patterns in the form of high-dose (“peaks”) and low-dose (“valleys”) regions. The aim is to capitalize the potential radiobiological advantages of inhomogeneous dose distributions and allowing for higher skin dose tolerance and increased depth dose to deep-seated tumors. A historical overview of the SFRT techniques has been reported by Yan et al. [27]. Initial clinical observations on the effectiveness and safety of SFRT utilizing megavoltage photon beams were presented by Mohiuddin’s group, particularly in palliative care for patients with bulky tumors intolerant to or resistant to conventional radiotherapy [28]. LRT was introduced in 2010 as a conceptual three-dimensional extension of SFRT with several uniquely different features. In lattice plans, a heterogeneous dose distribution is created within the planning target volume, forming a 3D array where high-dose regions (vertices) alternate with low-dose areas (periphery), resembling peaks and valleys. Despite delivering ablative doses to discrete sub-volumes, the valleys serve to minimize treatment-related toxicity. As for SFRT, the rationale behind LRT lies in the potential radiobiological advantages of highly inhomogeneous dose distributions. The high-dose regions within the tumor target aim to ablate or significantly debulk the tumor, while the lower-dose regions may stimulate an immune response or disrupt tumor vasculature. A review of the basic principles of LRT, together with technical recommendations and guidelines, has been recently proposed for its clinical implementation [29]. Two reviews focused on the application of LRT in clinical practice have been recently presented by Iori et al. [16] and Spałek [30], summarizing the available evidence on LRT, including its efficacy, safety, and potential applications in various tumor types. An updated review of the role of LRT in the present era of immunotherapy has been reported by McMillan et al. [31].

The largest clinical experience to date was presented in 2024 by clinicians at the Mayo Clinic, who reported the results of a retrospective cohort of 176 patients with 186 treated sites [32]. The most common LRT dose was 20 Gy in one fraction delivered with the VMAT technique. Median gross tumor volume was 480 cc (8-10898). Median follow-up was 322 days, with 1-year overall survival of 37% and 1-year local control of 81%. Equivalent uniform dose and mean dose were highly predictive of local control. Grade 3 toxicity occurred in nine patients.

The ongoing research and technological advancements discussed in these papers suggest a promising future for LRT in the treatment of cancer.

**Figure 1:**
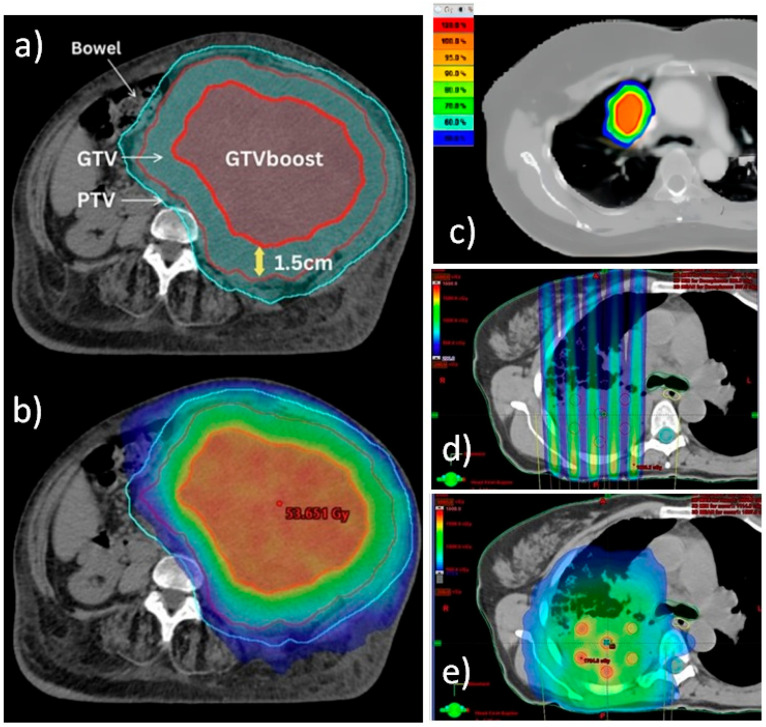
Comparison of dose distribution patterns of PART (**a**,**b**), SBRT (**c**), SFRT (**d**), and LRT (**e**) techniques. (**a**,**b**) Definition of target volumes and dose wash isodoses for PART planning for a bulky unresectable sarcoma. Courtesy of reference [9]. (**a**) Choice of authors for targets delineation. Diagnostic CT/MRI or PET/CT were fused with CT planning and the GTV was contoured. Next, a 1.5 cm isotropic contraction from the GTV was applied to form the GTV boost inside the GTV. Then, a 1 cm isotropic expansion from GTV was applied to generate the PTV. (**b**) Relative dose distribution prescribing 20 Gy to PTV (blue isodose) and 50 Gy to GTV boost (red isodose). (**c**) Typical dose distribution for an SBRT treatment of a lung lesion. Courtesy of reference [33]. Isodoses range from 50% (blue) to 100% (red) of dose prescription. (**d**,**e**) Irradiation of a bulky lung tumor. Courtesy of reference [27]. (**d**) Typical non-uniform radiation dose distribution of SFRT planning across the target volume, using grids to create spatially fractionated dose patterns in the form of high-dose and low-dose regions. Note that for deep-seated bulky tumors, SFRT faces the problem of high doses falling into normal tissue. (**e**) Typical dose distribution of LRT irradiation, where the creation of multiple localized high-dose small spheres called vertices with a certain degree of separation within the tumor volume is clearly visible. This technique also allows the dose level to be kept lower in the periphery of the tumor to avoid related toxicity.

To better highlight the spatial aspects of the different techniques, Figure 1 presents the dose distribution patterns of PART, SBRT, SFRT, and LRT for different cases. These representative cases are taken from the literature [9,27,33].

## 8. Limitations of This Review

Though there are promising preliminary data in terms of efficacy and safety and some impressive results in terms of treatment response, the evidence reported by this review has different limitations. Firstly, with respect to efficacy, the quality of evidence on PART is still low, as many publications were case reports or case series with small samples, different irradiation schedules, and different primary endpoints, all acting as potential confounders. In addition, the heterogeneity in target definition for PART is a significant challenge that needs to be addressed to improve the consistency and effectiveness of this treatment modality. Functional imaging is therefore essential in the implementation of this technique for a robust definition of the most aggressive or symptomatic tumor areas needing a dose intensification. Current data do not allow a direct comparison with standard radiotherapy in terms of survival, local recurrence, or quality of life, and all these endpoints deserve further investigation.

Several challenges and unanswered questions remain. Further research is crucial to fully elucidate the long-term efficacy and toxicity of PART, compare its outcomes with other treatment modalities, and identify optimal patient selection criteria. While early results are encouraging, larger-scale, prospective studies are needed to solidify the role of PART in the management of bulky tumors. Also, the role of PART as potential neoadjuvant therapy aiming to improve surgical outcomes needs to be investigated.

## 9. Conclusions

This narrative review presents a synthesis of the available data and serves as a basis for further analysis. PART has emerged as a promising treatment modality for bulky tumors, offering a potential paradigm shift in cancer care. By strategically delivering high doses of radiation to the most aggressive portions of the tumor while sparing surrounding healthy tissues, PART aims to maximize tumor control while minimizing treatment-related toxicity. This approach for large unresectable tumors may represent a further step forward over conventional palliative radiotherapy techniques, providing preliminary satisfactory symptomatic response, pain relief, tumor debulking, and local control.

Investigating the potential of combining PART with other treatments, such as chemotherapy or immunotherapy, could further enhance its therapeutic impact. Additionally, exploring the use of predictive biomarkers could personalize treatment strategies and improve patient outcomes. As our understanding of tumor biology and treatment response evolves, PART is poised to play an increasingly important role in the fight against cancer.

## Figures and Tables

**Table 1:** Summary of literature on clinical evidence. All doses are expressed as physical doses (Gy), except the study of Nomiya et al. (2014) [13], where doses are converted to equivalent dose at 2 Gy per fraction (EQD2). Legend: OS: overall survival; LC: local control; fr: fractions; GTV: gross tumor volume; FFLP: freedom from local progression; FFDP: freedom from disease progression.

First Author, Year	Tumor	Patients	Dose	Tumor Dimension	Main Findings
Bai, 2018 [2]	Lung	30	5–9 Gy in 3–6 fr to GTV	>4.5 cm	2-year LC and OS was 85.7% and 55.6%, 36.7% had partial response
Tubin, 2019 [3]	Lung	20	10–12 Gy in 1–3 fr	6–17 cm	1-year cancer specific survival was 90%
Wittgenstein, 2023 [4]	Head–neck	21	5 × 10 Gy or 10 × 3 Gy + 10 × 6 Gy	49–1179 cm^3^	No side effects
Medici, 2023 [6]	Sarcoma	1	40 Gy in 4 fr to GTV	no data	Complete metabolic response
Nomiya, 2015 [8]	Sarcoma	1	96 Gy in 33 fr to GTV	12 × 16 × 16 cm	Tumor reduction of 80% following resection
Yu, 2024 [9]	Sarcoma	18	50 Gy in 5 fr	>5 cm	89% of patients with partial response, mean tumor reduction of 49.5%, 1-year OS, FFLP, and FFDP of 61%, 83%, and 35%
Lin, 2016 [10]	Hepatocellular	1	57.2 Gy/26 fr	12.5 cm	Tumor reduction of 89%
Chen, 2023 [12]	Urinary tract	26	40–58 Gy/15–26/15–32 Gy	>4 cm	1-year LC and OS of 83.2% and 72.2%, no grade ≥ 3 toxicity
Nomiya, 2015 [11]	Breast	3	110–140 Gy	>15 cm	100% complete response, no grade ≥ 3 toxicity
Nomiya, 2014 [13]	Rectum	1	77 Gy EQD2	15 cm	Disappearance of tumor after 4 months

## Data Availability

No new data were created or analyzed in this study. Data sharing is not applicable to this article.

## Abbreviations

The following abbreviations are used in this manuscript.

PART	partial ablative radiotherapy
SBRT	stereotactic body radiation therapy
IMRT	intensity-modulated arc therapy
VMAT	volumetric modulated arc therapy
